# The Certified QPR Pathfinder Training Program: A Description of a Novel Public Health Gatekeeper Training Program to Mitigate Suicidal Ideation and Suicide Deaths

**DOI:** 10.1007/s10935-023-00748-w

**Published:** 2023-09-23

**Authors:** Paul G. Quinnett

**Affiliations:** 1 Department of Psychiatry and Behavioral Sciences, University of Washington School of Medicine, Seattle, WA USA; 2 The QPR Institute, Inc., P.O. Box 2867, Spokane, WA 99220 USA

**Keywords:** Gatekeeper training, Suicide prevention, Training, Health services

## Abstract

Suicide represents a significant public health concern. One approach to reducing suicide rates is to train gatekeepers—community members who, through their day-to-day practices, interact with a substantial proportion of the population—to detect individuals at elevated suicide risk and refer them to appropriate mental health care services. One of the most well-known community gatekeeper training programs is Question, Persuade, and Refer (QPR), which has been delivered to millions worldwide. Gatekeeper training, including QPR, shows considerable promise in reducing suicide risk. However, one limitation of existing gatekeeper training programs is that they rely on referrals to mental health services, which are often non-existent, understaffed, and/or undertrained regarding suicide risk. As such, novel approaches are needed to equip community gatekeepers with primary mental health first aid and suicide-focused counseling. This article describes, for the first time, the fundamental concepts of a newly developed and more expansive version of QPR, the QPR Pathfinder Training. The QPR Pathfinder Training is web-based training program designed to create a cadre of “super gatekeepers” to address suicide at scale. The QPR Pathfinder Training will equip communities to address the critical shortage of mental health care services around the globe and, in turn, reduce mental health morbidities and decrease the suicide rates.

Each year, nearly 50,000 individuals in the United States and 800,000 individuals worldwide die by suicide (Centers for Disease Control and Prevention [CDC], 2022; Naghavi, 2019). Tens if not hundreds of millions more individuals around the globe seriously think about suicide annually (Nock et al., 2008). Connecting suicidal individuals to mental health services remains a critical suicide prevention approach (US Department of Health and Human Services Office of the Surgeon General and National Action Alliance for Suicide Prevention, 2012). However, few individuals experiencing suicidal ideation report accessing mental health services (Hom et al., 2015; Hom & Stanley, 2021; Stanley et al., 2015), suggesting that innovative and scalable solutions are needed to provide services to at-risk individuals.

Gatekeepers are well-positioned to address this need. Community gatekeepers are people in a strategic position who are trained to identify others experiencing suicidality (Hawgood et al., 2021; US Department of Health and Human Services Office of the Surgeon General and National Action Alliance for Suicide Prevention, 2012). Examples of gatekeepers include teachers, student peers, military personnel, primary care physicians, and non-clinical administrative staff embedded in medical settings (Burnette et al., 2015). Most commonly, after identifying at-risk individuals, gatekeepers are taught to make referrals to a mental health professional for assessment and care.

Programs that train community gatekeepers show considerable promise in reducing suicide risk. In their systematic review, Isaac et al. (2009) found that gatekeeper training is associated with significant changes in trainees’ knowledge, beliefs, and attitudes regarding suicide prevention. Initial evidence, too, shows that gatekeeper training yields increases in practical suicide prevention skills among its recipients (Morton et al., 2021). Although more programmatic development and rigorous research is needed to determine the full promise of gatekeeper training programs, including the degree to which gatekeeper training may reduce the suicide death rate (Burnette et al., 2015; Mann et al., 2021), training community gatekeepers appears to be a promising suicide prevention strategy (Hom et al., 2015). In short, and consistent with the three-step theory of suicide (Klonsky et al., 2021; Klonsky & May, 2015), the gatekeeper intervention is likely to decrease suicide risk through (1) reductions in psychological pain and hopelessness, (2) establishing or re-establishing social connectedness, and (3) slowing or diverting the journey to a suicide attempt while enhancing resiliency.

One of the most well-known gatekeeper training models is Question, Persuade, and Refer (QPR; Quinnett, 1995), a 90-minute program traditionally conducted in-person. In QPR, trainees are taught to recognize individuals exhibiting suicide warning signs—“the earliest detectable sign that indicates heightened risk for suicide in the near-term (i.e., within minutes, hours, or days)” (Rudd et al., 2006). Once an individual is identified as potentially at-risk for suicide, the QPR program trains gatekeepers to take a non-judgmental stance that instills hope. Indeed, the primary action of the QPR Pathfinder Training is to reduce immediate risks for suicide. As appropriate, but not always, this action involves connecting the at-risk individual to appropriate mental health care resources. Initially, the QPR program was administered by a network of trained personnel worldwide; the training now also occurs via a web-based portal. It is important to note that QPR—like most, if not all, community gatekeeper training programs—is not intended to be a substitute for traditional clinical care. Multiple studies have shown that the QPR program is effective in increasing suicide prevention knowledge, attitudes, skills, and behaviors (Aldrich et al., 2018; Litteken & Sale, 2018; Mitchell et al., 2013; Wyman et al., 2008), One study found that the implementation of the Garrett Lee Smith (GLS) suicide prevention program, of which QPR gatekeeper training is a core component, was associated with a significant reduction in the suicide rates of individuals aged 10–24 years (Walrath et al., 2015).

In 2019 alone, the QPR Institute trained approximately 500,000 gatekeepers to recognize and refer people experiencing suicidal ideation and urges to providers. Other organizations trained thousands more gatekeepers. Busy hotlines referred even more thousands to professional services. In a word, case-finding capacity is growing while competent referral resources remain limited or stagnant. Indeed, although a primary action of gatekeepers is to reduce imminent risks for suicide, the referral action of community gatekeepers can only be successful if competent and accessible mental health professionals are available to accept the person as a “new patient” in a timely manner. This “system of care” is often nonexistent in some communities and/or acknowledged as broken, inadequate, and sometimes harmful (Schmitz et al., 2012). While greater demand for competent care is rising, barriers remain that block the pipeline of qualified providers (Hom et al., 2015).

One solution to address these barriers is to create a new category of suicide first responders trained in the evidence-based knowledge and skills currently available (but largely unused) by healthcare professionals, thus creating a new workforce of community-based suicide crisis responders. Such an approach would expand on traditional forms of community gatekeeper models and enhance the skills necessary to mitigate suicide risk. This article describes the key concepts of a newly developed and more expansive version of QPR, the QPR Pathfinder Training, which is a web-based, highly scalable training program to develop a cadre of community gatekeepers focused on addressing the public health issue of suicide. Specifically, this article provides a conceptual overview of the program, as well as pragmatic implementation considerations. This article is intentionally descriptive in nature. The QPR Pathfinder Training is rooted in a strong empirical base, including its predecessor QPR (Aldrich et al., 2018; Litteken & Sale, 2018; Mitchell et al., 2013; Walrath et al., 2015; Wyman et al., 2008). However, prospective data on the QPR Pathfinder Training adaptation are forthcoming; this article provides a foundational grounding from which empirical studies will follow.

## Conceptual Overview of the QPR Pathfinder Training

QPR Pathfinder Training is an online, evidence-based, competency-focused, accessible, and affordable training solution that can be built and delivered at scale to communities anywhere broadband is available that will directly target and benefit those at risk of suicidal ideation, attempts, and death. This training program teaches specific knowledge, skills, and attitudes to tackle suicidal ideation. Indeed, social cognitive theory posits that increases in the knowledge, beliefs, and confidence in enacting a behavior will lead to increases in the desired behavior (Bandura, 1991). In the case of suicide prevention gatekeeper training programs, training will impact each of these domains, leading to increases in asking about suicide, decreasing imminent risk for suicide, and referring to mental health services when indicated (Burnette et al., 2015).

Building upon existing training and skills of people in all helping professions, including willing lay adults, this training program provides them with the latest evidence-based interventions to immediately reduce suicide risk and sustain a continuous risk reduction program for people struggling with whether to live or die. In addition to basic gatekeeper knowledge, skills, and attitudes, this new category of gatekeepers would be much better skilled in how to identify, engage with, and carry out immediate risk mitigation best practices endorsed by the Zero Suicide initiative (Brodsky et al., 2018), such as reducing access to means, collaboratively developing safety plans, and conducting follow-up caring contacts.

Just as first responders learn CPR to expand their range of helpful interventions, with more rigorous training, Certified QPR Pathfinders could become the go-to people in their communities when someone in a suicide crisis is identified. Also, this expanded training and role definition could be offered to all existing gatekeepers currently serving in only a “recognize, respond, and refer” role (e.g., school health professionals, law enforcement, clergy, and all health care providers). Moreover, by delivering the training online, trainees are, in turn, taught to provide essential emotional support and psychological first aid to people in remote areas.

In general, these “super gatekeepers” would be willing and able to be trained in next-level skills to render the equivalent of mental health first aid and basic suicide-focused counseling to at-risk persons. As QPR-trained pathfinders, they would willingly take on the task of assisting people during and after a suicide crisis, inclusive of helping people deal with suicidal ideation. With attention to training fidelity and standards, training would be customized to be acceptable to local norms and expectations. Mental health providers might welcome the opportunity to shift some of the suicide care necessary for patient safety to this trained and certified workforce, just as medical professionals eventually welcomed the emergence of CPR-trained pre-hospital emergency medical technicians as a new kind of professional.

Essentially, pathfinders would be the recipients of “task transfer training” in which professional-level knowledge and basic assessment and counseling skills would be taught to a predetermined level of demonstrable competency, followed by an exam-based certification process (Burnette et al., 2015). As an example, nurses are trained in how to give an injection in nursing school and, in turn, teach their diabetic patients to do the same. Effective task transfer training is done all the time (including training more people to give vaccinations in response to the COVID-19 pandemic), and there is no reason not to do it in suicide prevention.

## Pragmatic Considerations of the QPR Pathfinder Training

### Who are Potential Pathfinders?

Pathfinder training would be suitable for “any willing heart” and would include community members who fit the “natural helper” or “healer” or “curandero” role where they live, work, play, and pray. Experience has shown that saving lives from suicide is its own reward and finding people to fill this new category of job should not be difficult. The success of peer support programs for various groups, including youth, is well documented (Mutschler et al., 2022; Shalaby & Agyapong, 2020; White et al., 2020). Pathfinders must be at least 17 years old; there are no other exclusions.

The first rank of such a potential suicide prevention workforce already exists: mental health and substance use disorder peer support professionals. This workforce goes by many names, including peer support workers, peer coaches, peer recovery coaches, peer advocates, and peer recovery support specialists (Videka et al., 2019). As a group, they are already well-informed about populations known to be at elevated risk of suicide morbidity and mortality. Peer support professionals already have training in mental health literacy, substance use disorders, basic counseling skills, and enjoy a robust evidence base to support their efficacy (Videka et al., 2019). They enjoy existing infrastructure, professional status, a credentialing process, and an accepted state and federal service reimbursement system. Many bring the benefit of lived experience and personal recovery. Training as a pathfinder would add an essential credential to their current skill set.

A second rank of potential pathfinders are those who can be recruited from basic QPR training as delivered online and face-to-face to more than 30,000 adults per month in the US and abroad. While stepping up to help people experiencing suicidal ideation and urges in their community might not appeal to many, if even a tiny percentage of those trained as basic gatekeepers stepped up to become pathfinders, the additional training and certification might lead to further training in peer support training and a rewarding new career. It is unknown how those trained in basic QPR might become a pipeline of recruits for this work as pathfinders, but many ask for additional training in how to be helpful to suicidal people. These recruits can also be successfully trained in basic counseling skills to provide essential and effective psychotherapy where no clinical providers are available (Chibanda et al., 2015).

### How is the QPR Pathfinder Training Delivered?

Delivered online using the latest e-learning technologies, Certified QPR Pathfinders training is scalable and deliverable worldwide. QPR Pathfinder Training leveraged a new e-learning technology called Mazetec (2022). Mazetec is a learning management system that allows learners to acquire knowledge and skills from the active learning process of interacting directly with the required content in unique ways to construct their competencies. Training is deliverable on any PC or mobile device and was engineered for users with low bandwidth, thus knocking down the brick-and-mortar costs of travel and traditional classroom suicide prevention and intervention training, even in remote areas where only satellite internet is available.

The interactive curriculum is delivered over three days, or about 14–20 h. The QPR Institute has provided online extended training programs for a global audience for more than 15 years, including basic counseling and risk mitigation interventions. Learner satisfaction with training is high. The curriculum is taught by four experienced, well-published, expert clinicians—two clinical psychologists and two psychiatrists.

Pathfinder training content can be customized for specific groups, languages, urban, rural, and indigenous audiences. Indeed, the QPR gatekeeper training program has been customized for several countries and has been translated into more than a dozen languages. In sum, basic QPR travels well, and there is no reason to believe QPR Pathfinder Training would not do the same.

### What does the QPR Pathfinder Training Entail?

The QPR Pathfinder Training consists of four programs that range from 14 to 20 h. One for those working with youth and young adults, one for those working with adults and older adults, one lifespan edition, and one veterans and military edition. Core and overlapping content areas include 21 modules, while additional modules are included in the youth and young adult edition, and additional modules are required for the adult and older adult edition, as well as the lifespan and military/veteran editions. Interested students can complete all modules if desired.

**Fig. 1:**
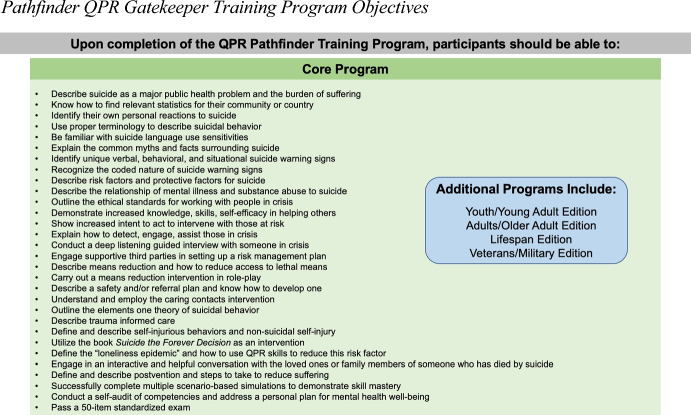
Pathfinder QPR gatekeeper training program objectives

We list in Fig. 1 the QPR Pathfinder Training goals as expected competencies (Hawgood et al., 2021). To develop the training content, we consulted and followed guidelines published by a Task Force of the National Action Alliance for Suicide Prevention (2014). This includes information about personal reactions, respect, confidentiality, risk and protective factors, screening, open and direct talk about suicide, safety planning, access to lethal means, and others. Treatment and scope of practice issues for various professions are not included as pathfinders are not necessarily mental health professionals. While pathfinders are not trained in a formal suicide risk assessment method per se, they are trained to assess the full scope of risk and protective factors within the context of trauma-informed care, and the other topics recommended by the task force.

The QPR Pathfinder Training is based on the *active learning model*. Using real-world simulations and a scenario-based learning environment, pathfinder students are obliged to look at a problem or question and make a decision. Reflection, a key element in learning and consolidation of long-term memory, is required at every choice point. Immediate, customized feedback is provided on each decision (correct or incorrect), sometimes with a brief mini lesson authored by the course mentor or coach. Errors are expected. Participants may make as many attempts as needed to complete each learning task, thus enabling a 100% score on all modules.

The simulation-based learning experience intends to teach through interactivity, in “engage–fail–learn” or “test to teach” mode. This type of active learning has been favorably examined and produces superior learning outcomes (Koedinger et al., 2016). While the course includes some reading or passive watching of a video lecture, context exams follow each module.

In the Mazetec format, the learner is presented with, as an example, a person in crisis with several options. Each option has consequences. The learner’s selected response may end the lesson, warn the learner as to the nature of their erroneous choice, and loop them back to the previous question or move them on to the next step in the simulation and the next set of possible responses. Using branching logic, learners can be decoyed down a decision tree until an adverse outcome occurs, then returned to the choice point where the error was made and given the option of another way forward.

In the active learning approach used in pathfinder certification training program, the learner makes hundreds of decisions to master the content, both in knowledge acquisition and in the application of that knowledge and skill to real-world scenarios where advanced QPR Pathfinder Training skills must be demonstrated to succeed. Each of dozens of learning and practice mazes must be completed successfully (100% compliance) for the learner to move forward and complete the certification training. The certification process also involves the successful completion of a 50-item standardized exam.

Finally, the Mazetec software data collection system and analytics capture every student’s keystroke, time to decide, choices made, and other information to enable a comprehensive record of the applicant’s knowledge and skill mastery. As part of program evaluation efforts, the QPR Institute will evaluate outcomes in several domains, including knowledge, attitudes, skills, abilities, and self-efficacy in working with those at risk of suicide. This data provides an individual record and performance-based evaluation of Certified QPR Pathfinders, as well as inputs for program improvements.

Some training simulations include a countdown timer to add a sense of urgency or conflict in, say, the recognition of a fleeting suicide warning sign. This gamified approach may take learners into wrong decision trees leading to adverse outcomes (e.g., making a wrong choice during an intervention can lead to a suicide attempt). Many jobs require making difficult decisions under uncertainty and time pressure, but few of them require making decisions upon which a life depends. In responding to acute suicide crises, time pressure is often very real. QPR Pathfinding training includes training under pressure.

### Where is the QPR Pathfinder Training Implemented?

The predecessor to QPR Pathfinder Training, QPR, is implemented globally, including in major Fortune 500 companies, 4,000 colleges and universities, thousands of secondary schools, most state departments of health or mental health, the construction industry, and federal agencies (e.g., US TSA and FEC). Thus far, 6 million gatekeepers have been trained by 16,000 active instructors, with an additional 300 new instructors added per month. The QPR Pathfinder Training is positioned to tap into this vast network. The QPR program was quickly and widely scaled due to its demonstrated evidence base. As evidence accumulates for the QPR Pathfinder Training adaptation, it is anticipated that a similarly widespread gatekeeper training program will be available.

## Continuing Education and Skills Refresher Training

Once all training requirements are met and a certificate is awarded, the QPR Pathfinder will be registered in an international database and registry, thus allowing the institute to stay in contact with them.

However, it is important to note that multiple studies have shown that the basic QPR gatekeeper training impacts on knowledge, skills, and attitudes diminish over the months and years following initial training (Cross et al., 2011; Matthieu et al., 2008; Tompkins et al., 2010). This decay in knowledge and skills may negatively impact gatekeeper readiness to conduct a competent intervention quickly and effectively. However, recent research found that the combination of a role-play during the index QPR training session, followed by a low-cost emailed text-only online role-play booster session at six months produced a significantly larger proportion of gatekeeper identifications, referrals, and notifications of the referral source (Godoy Garraza et al., 2021). The authors conclude that gatekeeper training can thus be enhanced through active learning strategies.

**Fig. 2:**
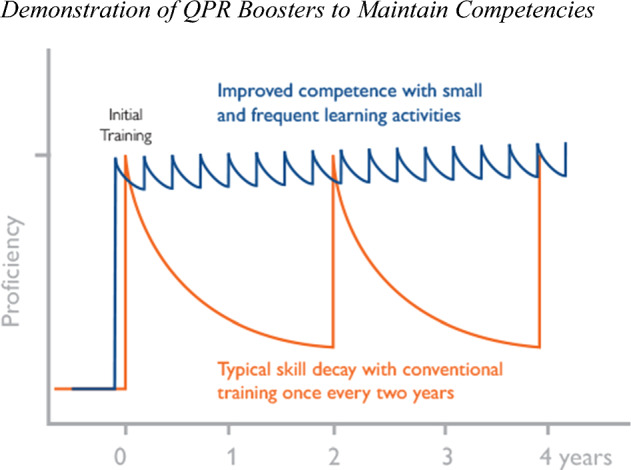
Demonstration of QPR Boosters to maintain competencies. *Note *Proficiency (y-axis) refers to trainees’ degree of competency in the skills taught during the QPR Boosters. The number of years (x-axis) refers to the time since the index training in the QPR Pathfinder Training

In light of this research and to address post-training diminishment in the knowledge, skills, and attitudes necessary to carry out safe and effective interventions, the training program includes research-to-practice QPR Boosters, a low-dose, high-frequency series of online booster sessions in the format of micro-lessons (2 to 10 min long) specifically designed to maintain the competencies taught in the Certified QPR Pathfinder Training (Fig. 2).

*QPR Boosters* modules are emailed to QPR Pathfinders at a minimum rate of six times per year for each 2-year interval of certification. Individual tracking of performance in these booster session training modules are compiled and available to the learner and/or their employer, thus allowing for any compliance training requirements that may develop. Using a blend of brief training review modules—and especially scenario-based, interactive real-world simulations—the QPR Institute aims to reinforce the confidence and competence of Pathfinders in the knowledge and skills they acquired during initial training.

## Competency-Based Training

The online training and examinations required to complete the QPR Pathfinder Training and earn a certificate have been carefully evaluated and designed to meet the recommendations set forth by the American Psychological Association’s Task Force on Assessment of Competence in Professional Psychology (Kaslow et al., 2007). This Task Force set forth 15 principles applicable to the education, training, and credentialing of professional and practicing psychologists over their career life span. Adopting these principles for this online training program and its recertification requirements reinforces and supports a “culture of competence” for QPR Pathfinders.

Specifically, this training program incorporates evidence-based and culturally competent practices and specifically addresses a series of developmental learning steps to include knowledge, attitudes, skills, self-perceptions, beliefs, and dispositions necessary to work effectively with people experiencing suicidal ideation and urges. The training includes scenario-based learning, “thought experiments” using multiple vignettes to assess critical thinking, judgment, emotional and interpersonal intelligence, as well as the capacity to engage and successfully assist people at risk.

The evidence-based suicide risk mitigation methods taught in this program are identical to those taught to clinical professionals but are culturally adapted to be accepted at the community level. Therefore, a high degree of fidelity exists between the foundational competencies expected of professionals interacting with those at risk and those of successful Pathfinder trainees who earn a certificate.

Evaluations of competence in the QPR Pathfinder Training include multi-trait, multi-method, and multi-informant processes. More than one evaluation methodology is used, including online quizzes, online simulated role-plays and collective feedback from other students, as well as the collection of individual performance metrics and analysis as measured against expert benchmarked standards.

Self-reflection and self-assessment modules are included to address the learner’s limits of expertise and the need for additional training or experience.

As this training program is intended to train learners to deal with persons coping with end-of-life decisions, it clearly fits the Institute of Medicine’s description of practice as a “moral enterprise.” Therefore, a training module devoted to ethics in human services as a cross-cutting competency is addressed in lecture, reading, and an ethics-specific scenario-based examination, including items related to helping services delivered over the internet. A self-care module is included as well.
